# Pulmonary involvement of ANCA-associated vasculitis in adult Chinese patients

**DOI:** 10.1186/s12890-022-01829-y

**Published:** 2022-01-12

**Authors:** Peining Zhou, Zhiying Li, Li Gao, Chengli Que, Haichao Li, Jing Ma, Guangfa Wang, Min Chen

**Affiliations:** 1 Department of Respiratory and Critical Care Medicine, Peking University First Hospital, 8 Xishiku Street, Xicheng District, Beijing, 100034 China; 2 Department of Nephrology, Peking University First Hospital, Beijing, China; 3 Department of Radiology, Peking University First Hospital, Beijing, China

**Keywords:** ANCA-Associated vasculitis, Pulmonary involvement, Mortality, Outcome

## Abstract

**Objective:**

The aim of this study was to clarify the clinical characteristics and long-term outcomes of ANCA-associated vasculitis (AAV) patients with pulmonary involvement from a single Chinese cohort.

**Methods:**

Newly diagnosed AAV patients with pulmonary involvement, as defined by CT, were recruited from January 2010 to June 2020. Clinical data and CT images were collected retrospectively. Baseline CTs were evaluated and re-classified into four categories: interstitial lung disease (ILD), airway involvement (AI), alveolar hemorrhage (AH), and pulmonary granuloma (PG).

**Results:**

A total of 719 patients were newly diagnosed with AAV, 366 (50.9%) of whom combined with pulmonary involvement at baseline. Among the AAV cases with pulmonary involvement, 55.7% (204/366) had ILD, 16.7% (61/366) had AI alone, 14.8% (54/366) had PG, and 12.8% (47/366) had AH alone. During follow-up of a median duration of 42.0 months, 66/366 (18.0%) patients died, mainly died from infections. Survival, relapse, and infection were all significantly different based on the radiological features. Specifically, the ILD group tends to have a poor long-term prognosis, the PG group is prone to relapse, and the AI group is apt to infection. The AH group has a high risk of both early infection and relapse, thus a poor short-term prognosis.

**Conclusion:**

AAV patients with diverse radiological features have different clinical characteristics and outcomes. Therefore, the intensity of immunosuppressive therapy must be carefully valued by considering the baseline CT findings among AAV patients with pulmonary involvement.

**Supplementary Information:**

The online version contains supplementary material available at 10.1186/s12890-022-01829-y.

## Background

Antineutrophil cytoplasmic antibody (ANCA)-associated vasculitis (AAV) is a group of disorders characterized by necrotizing inflammation of small- and medium-sized blood vessels and the presence of circulating ANCA. Clinical disease phenotypes include granulomatosis with polyangiitis (GPA), microscopic polyangiitis (MPA), and eosinophilic granulomatosis with polyangiitis (EGPA) [1]. Pauci-immune glomerulonephritis is a seldom type of circulating ANCA-negative AAV. Ethnicity difference was known as MPO-ANCA is predominantly among Asian patients with both GPA and MPA [2].

Pulmonary involvement is frequent and prominent among various manifestations of AAV. Radiologic finding, mainly computed tomography (CT), is key for the identification of pulmonary involvement. Previous studies showed that 52–80% of patients with AAV had pulmonary abnormalities on chest CT [3–5], mainly including interstitial lung disease (ILD), granuloma (nodules, masses, with or without cavitation), alveolar hemorrhage (AH). In most publications, ILD was defined as usual interstitial pneumonia (UIP) or non-specific interstitial pneumonia (NSIP) according to radiologic patterns [6, 7]. MPA is the main AAV subtype associated with ILD [4, 8–10]. Granuloma is more prevalent in GPA [4, 11]. The incidence of AH is 7–45% in GPA and 10–30% in MPA but is rare in EGPA [12, 13]. Airway involvement (AI) has been reported by some small size of cohorts [11]. However, there is still limited information focusing attention on the association between different radiologic patterns and long-term outcomes among AAV patients with pulmonary involvement.

On this ground, the aim of this study was to further characterize the relationship between the clinical features and long-term outcomes according to the pulmonary radiologic patterns in a retrospective cohort spanning 10 years with 366 adult Chinese AAV patients with pulmonary involvement. Furthermore, we counted the relapses and infectious events based on different radiologic patterns and identified their predictors.

## Methods

### Study population

Seven hundred and nineteen patients newly diagnosed as AAV at the Peking University First Hospital, from January 2010 to June 2020, were screened retrospectively. Inclusion criteria were defined as follows: (1) patients fulfilled the Chapel Hill Consensus Conference definition for AAV and the entry criteria required by Watts’ algorithm [14, 15]. (2) patients with pulmonary involvement, as defined by chest CT. Exclusion criteria were defined as follows: (1) age younger than 18 years; (2) patients with secondary vasculitis or with comorbid autoimmune disease; (3) other causes known to induce CT abnormality; and (4) inadequate clinical data or without baseline CT.

This study was conducted in compliance with the Declaration of Helsinki principles and was approved by the Ethical Committee of Peking University First Hospital. Informed consent was not required for this work because it consists of retrospective data, and all treatment decisions were made prior to our evaluation.

### Evaluation of chest CT images

The baseline chest CT images were reviewed independently by two pulmonologists (P.Z and J.M) and one expert pulmonary radiologist (L.G) who were blinded to clinical and laboratory findings. All images were obtained using regular-dose CT.

According to CT images, we divided all patients into four groups: patients with ILD (the ILD group), patients with pulmonary granuloma (the PG group), patients with alveolar hemorrhage alone (the AH group), patients with airway involvement alone (the AI group). ILD and PG are generally considered to be the most common imaging findings in MPA and GPA, respectively, and these two categories of imaging were first distinguished in our cohort. If typical ILD manifestations, including honeycombing, reticulation, and ground-glass opacity (GGO), were predominant, they were assigned to the ILD group. Patients were assigned to the PG group if they presented with nodules, masses, with or without cavitation as the main manifestation, except for infection.

In this study, patients with alveolar hemorrhage or lower airway involvement as the only pulmonary manifestation were classified as AH group or AI group, respectively. The features of AH were regarded as bilateral diffuse GGO and consolidation on CT scan without alternative explanation. Patients with AH at onset and no CT abnormality remaining after treatment were also classified into the AH group. Airway involvement (AI) in this study was defined as involvement of lower respiratory airway including trachea and bronchus, and small airways without obvious mass or ILD. Small airway involvement was recorded when the manifestations of either centrilobular nodules(tree-in-bud) or air trapping patterns according to chest CT scans, except for secondary infection or other airway-related comorbidities.

To better exhibit the overlapped imaging findings presented in each subgroup, specific abnormal CT findings were further statistic according to Fleischer Society guidelines [16], including GGO, reticulation, honeycombing, nodules, masses, cavities, bronchial stenosis, bronchiectasis, tree-in-bud, air trapping, emphysema, pleural thickening. The classification of ILD in our study was confirmed after an independent review of the CT scans by an experienced pulmonologist (J.M) and a radiologist expert (L.G), strictly following the 2013 idiopathic interstitial pneumonias classification from the American Thoracic Society/European Respiratory Society Statement [17].

### Data collection and definitions

Data were collected from diagnosis and during follow-up until death or June 2020. All ANCA-negative vasculitis was pathologically diagnosed. Disease activity was assessed by the Birmingham Vasculitis Activity Score (BVAS) [18]. AH was defined as bilateral alveolar infiltrates on CT scans without alternative explanation, plus at least one of the following: unexplained hemoptysis, progressive decrease in hemoglobin or anemia, or bronchoscopy evidence of progressive hemorrhagic BAL [19], or the percentage of hemosiderin-laden macrophages ≥ 20% of the total amount of the alveolar macrophages in BAL fluid [20]. Respiratory failure was defined by an arterial oxygen tension (P_a_O_2_) of < 60 mmHg(8.0 kPa), with or without an arterial carbon dioxide tension (P_a_CO_2_) of > 50 mmHg(6.0 kPa) [21], when breathing atmospheric air at sea level under resting condition, or the need for any type of mechanical ventilation [22]. Central nervous system (CNS) involvement was defined as new-onset neurologic deficits plus abnormal radiological and cerebrospinal fluid findings without other explainable causes [23]. Infection was recorded if hospitalization was required. Infection was considered based on clinical manifestation, laboratory data, radiographic imaging, and response to antibiotics. Relapse was defined by the recurrence of symptoms of active vasculitis in any organ system after remission is achieved with other causes excluded.

### Statistical analyses

We used an analysis of variance (ANOVA) to assess differences among subject groups, and post hoc comparisons were made using the Bonferroni/Dunn test. Differences in qualitative results were compared using the chi-square test, and post hoc comparisons (Bonferroni correction) were performed to detect differences between the groups. Multivariable Cox regression analysis was performed to evaluate the predictors of infection, relapse, and mortality. Variables associated with a given outcome at a *p* value < 0.1 in Kaplan–Meier analysis (categorical variables) or univariable Cox regression (continuous variables) were entered into the final multivariable model. Results were expressed as hazard ratios with 95% confidence intervals. The difference was considered significant if the *p* value < 0.05. Analysis was performed with SPSS software (v 27.0; SPSS, Chicago, IL, USA).

## Results

### Clinical characteristics

**Fig. 1:**
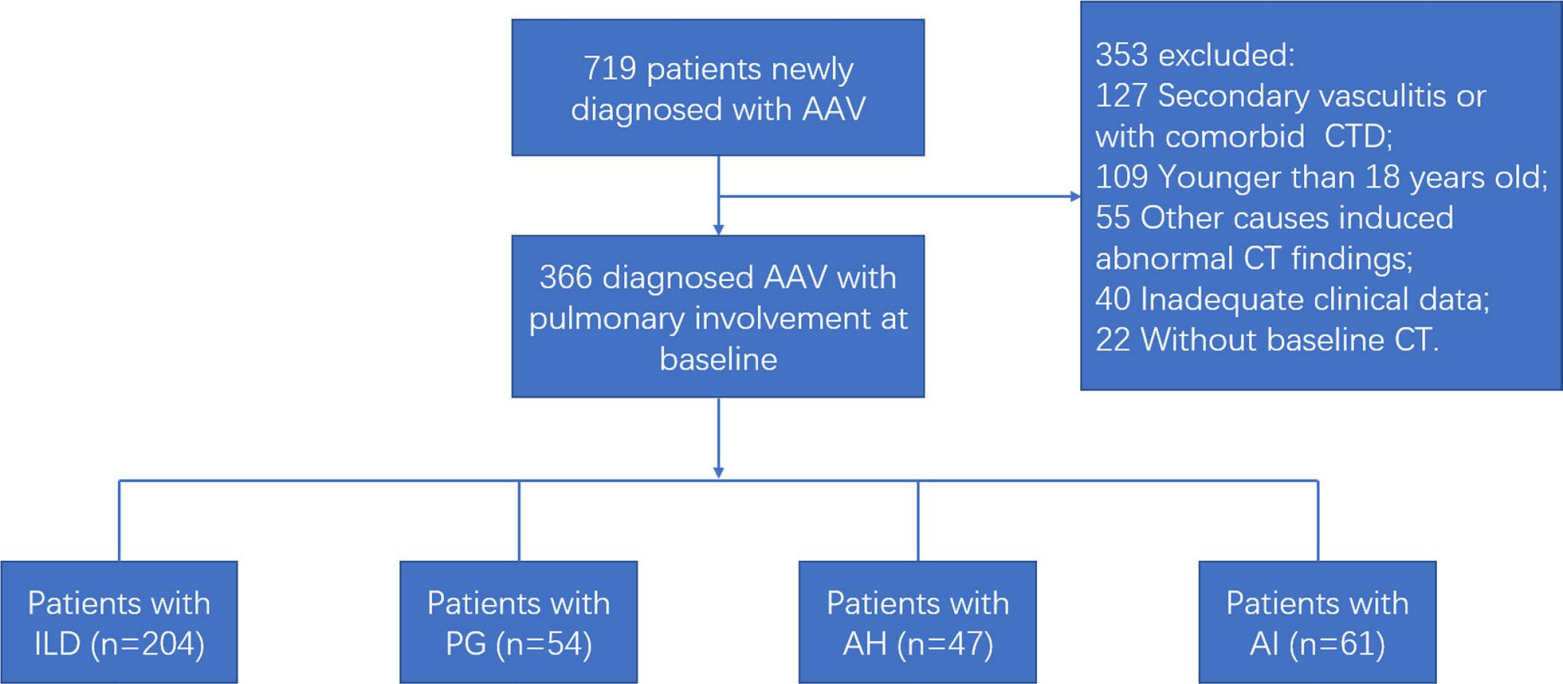
Flow chart of the study population. *AAV* ANCA-associated vasculitis, *AH* alveolar hemorrhage, *AI* airway involvement, *CT* computed tomography, *CTD* connective tissue disease, *ILD* interstitial lung disease, *PG* pulmonary granuloma

Three hundred and sixty-six patients newly diagnosed AAV with pulmonary involvement were eventually enrolled (Fig. 1), of which 249 patients were first admitted to the department of nephrology, 70 from the department of respiratory and critical care medicine, 43 from the department of rheumatology, 2 from the department of infectious disease, 1 from the department of neurology and 1 from the department of thoracic surgery. The mean age at onset was 66 [IQR 58–72] years old. Two hundred and ninety-nine (81.7%) patients were MPA, 60 (16.4%) were GPA and 7 (1.9%) were EGPA. Three hundred and fifty-eight (97.8%) patients were ANCA positive; 299(81.7%) were MPO-ANCA positive, 44 (12.0%) were PR3-ANCA positive, and 11 (3.0%) were double positive. We divided the AAV patients into four groups: the ILD group (n = 204), the PG group (n = 54), the AH group (n = 47), and the AI group (n = 61). Among the PG group, granulomatous lesions were confirmed by lung biopsy results in 17 (31.5%) patients and by renal biopsy findings in 25 (46.3%) patients.

The baseline clinical characteristics and laboratory findings are summarized in Table 1. The mean age was significantly higher in the ILD group compared with the other three groups. The incidence of cardiovascular complications was also higher in the ILD group compared to the PG group. The MPO-ANCA was predominant in all four groups. In the comparison among the four groups, the positive rate of MPO-ANCA was higher in the ILD and AI groups than in the AH and PG groups, and PR3-ANCA was detected more in the PG and AH groups than in the other two groups, respectively.

**Table 1:** Clinical characteristics and laboratory findings of patients with different radiological patterns

Characteristic	Alveolar hemorrhage (n = 47)	Interstitial lung disease (n = 204)	Pulmonary granuloma (n = 54)	Airway involvement (n = 61)
Age, median (IQR) (years)	56 (47–64)	68 (62–75)^d,e,f^	59 (53–69)	61 (58–68)
Sex (male), no. (%)	21 (44.7)	110 (53.9)	28 (51.9)	24 (39.3)
Ever smoker, no. (%)	13 (27.8)	85 (41.7)	19 (35.2)	17 (27.9)
MPA, no. (%)	36 (76.6)	187 (91.7)	25 (46.3)^d,g,h^	51 (83.6)
GPA, no. (%)	11 (23.4)	14 (6.9)^e^	26 (48.1)^d,g,h^	9 (14.8)
EGPA, no. (%)	0	3 (1.5)	3 (5.6)	1 (1.6)
Time of onset to diagnosis, median (IQR) (months)	1 (0–2)	2 (0–8)	1 (0–4)	1 (0–8)
Follow-up, median (IQR) (months)	39 (17–64)	40 (17–75)	52 (26–72)	42 (19–84)
*Comorbidities, no. (%)*				
Cardiovascular events^a^	18 (38.3)	112 (54.9)^d^	18 (33.3)	24 (39.3)
Malignancy	1 (2.1)	4 (2.0)	0	3 (4.9)
*Organ involvement at diagnosis, no. (%)*				
Fever	23 (48.9)	74 (36.3)	22 (40.7)	28 (45.9)
Weight loss (≥ 2 kg)	18 (38.3)	95 (46.6)	24 (44.4)	32 (52.5)
Cutaneous	5 (10.6)	9 (4.4)	5 (9.3)	2 (3.3)
Ear, nose and throat	14 (29.8)	57 (27.9)^d^	26 (48.1)	24 (39.3)
Eye	6 (12.8)	10 (4.9)^d^	10 (18.5)	7 (11.5)
Cardiovascular	2 (4.3)	4 (1.2)	1 (1.9)	2 (3.3)
Gastrointestinal	2 (4.3)	3 (1.5)	3 (5.6)	2 (3.3)
Central nervous system	3 (6.4)	5 (2.5)	4 (7.4)	3 (4.9)
Peripheral nervous system	3 (6.4)	34 (16.7)	10 (18.5)	13 (21.3)
Renal^b^	46 (97.9)	165 (80.9)	44 (81.5)	52 (85.2)
Renal insufficiency^c^	20 (42.6)	50 (24.5)	18 (33.3)	21 (34.4)
*Laboratory findings at diagnosis*				
MPO, no. (%)	33 (70.2)	179 (87.7)^d,e^	34 (63.0)	53 (86.9)^h^
PR3, no. (%)	11 (23.4)	12 (5.9)^d,e^	17 (31.5)	4 (6.6)^h^
Double positive, no. (%)	0	9 (4.4)	0	2 (3.3)
ANCA negative, no. (%)	2 (4.3)	3 (1.5)	2 (3.7)	1 (1.6)
Serum creatinine, mean ± SD, μmol/L	424.1 ± 312.9	323.6 ± 265.3	315.1 ± 249.4	398.0 ± 355.7
C-reactive protein, mean ± SD, mg/L	54.4 ± 53.9	45.9 ± 49.7	55.3 ± 52.0	45.1 ± 50.0
BVAS at onset, median (IQR)	20 (17–24)	17 (14–21)^d,e^	20 (15–25)	20 (14–24)
Five-Factor Score, no. (%)				
0	5 (10.6)	60 (29.4)	15 (27.8)	19 (31.1)
1	9 (19.1)	53 (26.0)	9 (16.7)	17 (27.9)
≥ 2	33 (70.2)^e,i^	91 (44.6)	28 (51.9)	25 (41.0)
*Bronchoalveolar lavage results at diagnosis*				
Lymphocytes count, mean ± SD	8.7 ± 8.0	9.4 ± 11.1	10.8 ± 14.8	5.8 ± 7.7
Lymphocytes > 20%, no. (%)	2/18 (11.1)	7/59 (11.9)	2/20 (10.0)	1/15 (6.7)
Neutrophils count, mean ± SD	43.9 ± 32.6	44.3 ± 30.7	32.8 ± 33.1	62.1 ± 31.0^ h^
Neutrophils > 5%, no. (%)	16/18 (88.9)	55/59 (93.2)	17/20 (85.0)	14/15 (93.3)
Hemosiderin-laden macrophages count, mean ± SD	60.2 ± 26.8	12.7 ± 20.4^d,e^	37.8 ± 31.7^ g,h^	5.1 ± 17.7^i^
Hemosiderin-laden macrophages ≥ 20%, no. (%)	16/18 (88.9)	15/59 (25.4)^d,e^	12/20 (60)	1/15 (6.7)^h,i^
*Pulmonary function test results at diagnosis*				
Obstruction, no. (%)	1/9 (11.1)	7/66 (10.6)^d^	9/19 (47.4)	4/12 (33.3)
Restriction, no. (%)	4/9 (44.4)	43/66 (65.2)	7/19 (36.8)	8/12 (66.7)
FEV1/FVC, mean ± SD	74.9 ± 9.3	80.0 ± 8.6^d,f^	70.1 ± 12.3	66.1 ± 14.0
FEV1% pred, mean ± SD	88.1 ± 27.7	83.4 ± 20.4	79.4 ± 21.8	70.1 ± 24.2
FVC% pred, mean ± SD	98.5 ± 27.3	79.6 ± 23.8^e^	86.9 ± 23.1	80.8 ± 14.7
TLC% pred, mean ± SD	94.7 ± 25.9	73.7 ± 17.6^e^	85.4 ± 19.5	79.5 ± 15.5
FEF 25–75% pred, mean ± SD	88.1 ± 4.0	62.8 ± 25.5	38.4 ± 30.3	23.78 ± 11.1^f,i^
FEF50% pred, mean ± SD	66.3 ± 33.6	75.3 ± 34.5^d^	50.0 ± 35.8	38.5 ± 21.8^f^
FEF25% pred, mean ± SD	54.5 ± 22.8	69.8 ± 44.5	45.4 ± 30.9	42.1 ± 18.7
DLCO % pred, mean ± SD	65.1 ± 28.8	51.6 ± 17.1	66.4 ± 21.9	57.3 ± 16.7
*Treatment, no. (%)*				
Induction				
Glucocorticoids pulses	32 (68.1)	78 (38.2)^e^	23 (42.6)	33 (54.1)
Immunosuppressant	41 (87.2)	147 (72.1)	37 (68.5)	47 (77.0)
Cyclophosphamide	38 (80.9)	142 (69.6)	35 (64.8)	45 (73.8)
Rituximab	0	1 (0.5)	1 (1.9)	0
Plasma exchanges	22 (46.8)	48 (23.5)^e^	17 (31.5)	18 (29.5)
Dialysis	21 (44.7)	50 (24.5)^e^	17 (31.5)	21 (34.4)
Maintenance				
Immunosuppressant	21/21 (100.0)	99/105 (94.3)	36/37 (97.3)	28/32 (87.5)
Cyclophosphamide	21/21 (100.0)	95/105 (90.5)	34/37 (91.9)	27/32 (84.3)
Rituximab	4/21 (19.0)	3/105 (2.9)^e^	5/37 (13.5)	2/32 (6.3)
Azathioprine	3/21 (14.3)	28/105 (26.7)	9/37 (24.3)	5/32 (15.6)
Mycophenolate mofetil	0	8/105 (7.6)	2/37 (5.4)	2/32 (6.3)

*AAV* ANCA-associated vasculitis, *AI* airway involvement, *ANCA* antineutrophil cytoplasm antibodies, *BVAS* Birmingham vasculitis activity scores, *DLCO* diffusing capacity for carbon monoxide, *EGPA* eosinophilic granulomatosis with polyangiitis, *FEF*_*25-75*_ forced expiratory flow at 25–75% of FVC, *FEF*_*50*_ forced expiratory flow at 50% of FVC, *FEF*_*25*_ forced expiratory flow at 50% of FVC, *FEV1* forced expiratory volume, *FVC* forced vital capacity, *GPA* granulomatosis with polyangiitis, *IQR* interquartile range, *MPA* microscopic polyangiitis, *no* number, *MPO* myeloperoxidase, *PR3* proteinase-3, *SD* standard deviation, *TLC* total lung capacity, *% pred* % predicted

^a^Cardiovascular events were defined as the presence of one or more of the following conditions at the time of AAV diagnosis: ischemic heart disease, congestive heart failure, hypertension, stroke, thromboembolism

^b^Renal involvement was defined as the presence of hematuria (≥ 10 red blood cells per high power field), proteinuria (≥ 500 mg/24 h), rise in serum creatinine > 30%, biopsy-proven glomerulonephritis, or need to initiate renal replacement therapy all attributable to active vasculitis

^c^Renal insufficiency was defined as serum creatinine level ≥ 500 μmol/L or undergoing dialysis

*p* < 0.05: ^d^ILD versus PG, ^e^ILD versus AH, ^f^ILD versus AI, ^g^PG versus AH, ^h^PG versus AI, ^i^AH versus AI

Patients in the PG group were more commonly associated with systemic AAV manifestations, such as ENT and eye symptoms compared to the ILD group. No significant difference regarding the prevalence of renal involvement and renal insufficiency between the four groups. The ILD group had a significantly lower BVAS compared to the PG and AH groups, and the AH group had a higher Five-Factor Score than the ILD and AI groups at baseline.

Bronchoalveolar lavage was performed in 112 (30.6%) patients, including 18 patients from the AH group, and BAL fluid hemosiderin-laden macrophage percentage was more than 20% in 16 (88.9%) patients from the AH group. The neutrophils count was significantly higher in the AI group compared to the PG group. The hemosiderin cells count was significantly higher in the AH group than the other three groups, and also higher in the PG group compared to the ILD and AI groups.

A total of 101 patients had pulmonary functional tests at baseline. The proportion of obstructive ventilation dysfunction was higher in the PG group compared to the ILD group. There were no significant differences in the proportion of restrictive ventilation dysfunction. However, the mean value of FVC% pred. and TLC% pred. were both lower in the ILD group than in the PG group. Intriguingly, surrogate markers of small airway disease, such as FEF_25–75_ and FEF_50_, were significantly decreased in the AI group. FEF_50_ was also decreased in the PG group compared to the ILD group.

There were no significant differences in the uses of immunosuppressors for induction therapy. However, the percentage of use of glucocorticoids pulse, plasma exchange, and dialysis was higher in the AH group than in the ILD group. The percentage of use of rituximab was also higher in the AH group than in the ILD group during maintenance therapy.

Ninety-nine (27.0%) patients occurred respiratory failure during follow-up, 63.6% (63/99) of whom from the ILD group, 8.1% (8/99) of whom from the PG group, 16.2% (16/99) from the AH group, and 12.1% (12/99) from the AI group.

### Prevalence of lung abnormalities on chest CT images

**Fig. 2:**
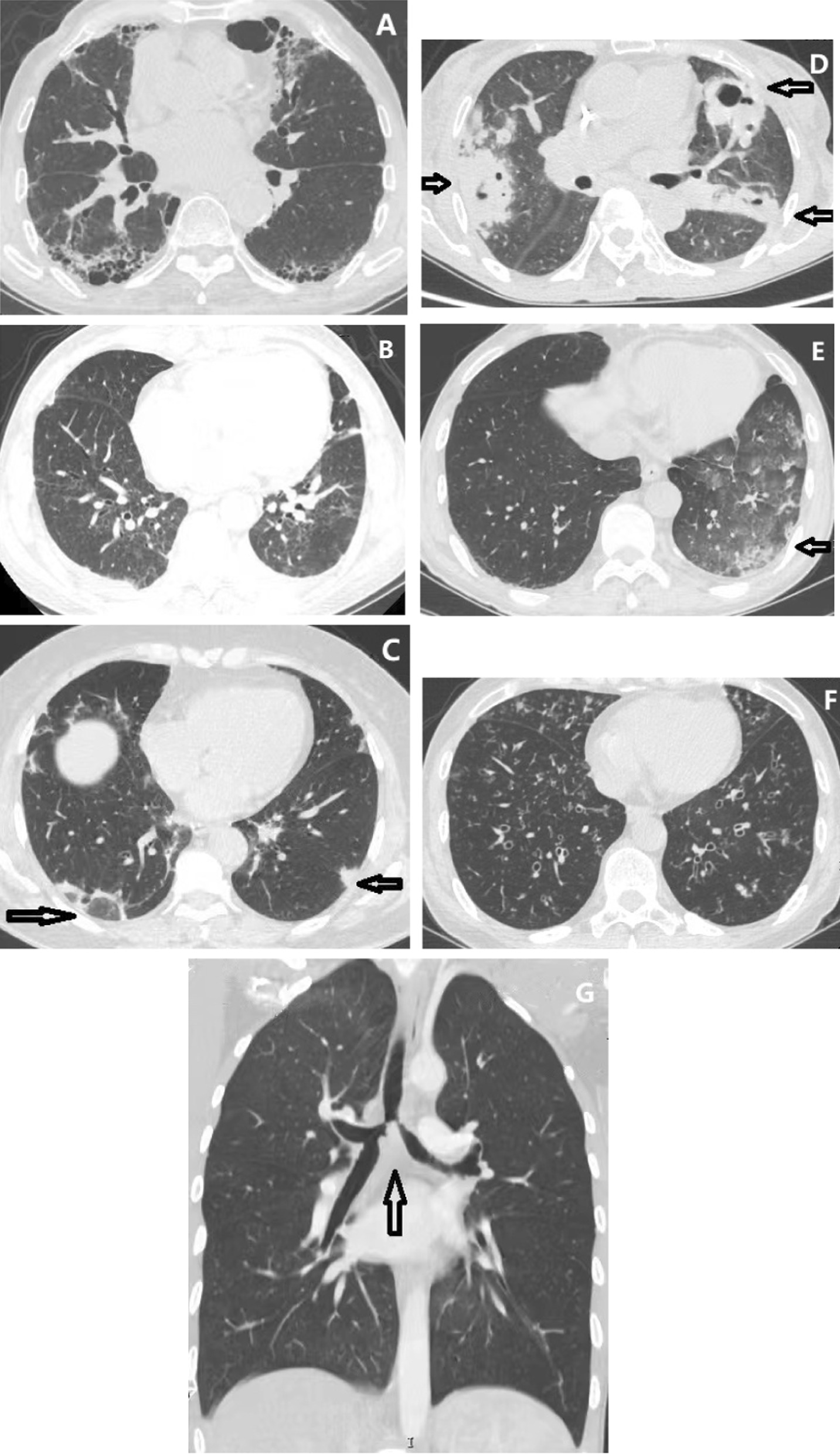
The representative computed tomography images of four groups. **a** usual interstitial pneumonia; **b** non-specific interstitial pneumonia; **c** organizing pneumonia; **d** pulmonary granuloma; **e** alveolar haemorrhage; **f** small airway involvement; **g** tracheobronchial stenosis. **a** An 81-year-old male came to the department of respiratory with progressive dyspnea and was found to be MPO-ANCA positive and diagnosed with MPA. The chest CT manifested fibrotic changes of UIP pattern in basal and subpleural predominance, traction bronchiectasis, and honeycombing. **b** A 56-year-old male came to the nephrology department with elevated serum creatinine on physical examination. He was MPO-ANCA positive and diagnosed with MPA. CT scan showed peripheral ground-glass opacity and typical subpleural sparing representing the NSIP pattern. **c** A 58-year-old female visited the respiratory department due to a persistent cough. She was MPO-ANCA positive and diagnosed as MPA. CT presented bilateral peribronchovascular and peripheral patchy consolidations combined with reversed halo sign, which is typical for OP. **d** A 65-year-old female came to the rheumatology department with multisystem involvement symptoms, including cough and hemoptysis. She was MPO-ANCA positive and diagnosed with GPA. CT showed bilateral nodules and consolidations, combined with cavitation. **e** A 47-year-old male visited the respiratory department due to hemoptysis and other multisystem symptoms. He was PR3-ANCA positive and diagnosed with GPA. CT demonstrated diffuse ground-glass attenuation in the left side. **f** A 58-year-old female visited the respiratory department with cough and sinusitis. She was both MPO-ANCA and PR3-ANCA positive and diagnosed with GPA. CT manifested as bilateral centrilobular nodules, regarded as the typical tree-in-bud sign. **g** A 27-year-old female was admitted to the respiratory department due to severe dyspnea. She was PR3-ANCA positive and diagnosed as GPA. CT showed tracheobronchial stenosis

The CT findings and comparison are summarized in Fig. 2a–d and Additional file 1: Table S1. Common CT abnormalities included ground-glass opacity (53.3%), honeycombing (39.9%), bronchiectasis (18.6%), air trapping (16.7%), bronchial stenosis (14.2%), reticulation (12.8%) and tree-in-bud (12.0%).

Comparing between four groups, honeycombing and reticulation were more frequent in the ILD group than in the other three groups. The PG group was manifested more frequently as nodules, masses, and cavities. Intriguingly, 8.4% (25/299) of MPA cases in our cohort manifested as PG pattern, 10 of whom were confirmed by lung biopsy and the remaining 15 patients by kidney biopsy. The CT characteristics included 19 (6.4%) cases with nodules, 6 (2.0%) cases with masses, and 5 (1.7%) cases combined with cavities, which are relatively rare and special imaging findings. In some previous studies focusing on images of patients with MPA, Suzuki et al. reported that 7% of MPA cases presented as nodules on CT scans [6], and Yamagata et al. reported that 9% of MPA patients with nodules [3]. A European study reported that nodules pattern was manifested in 16% of MPA, and 2% of whom combined with cavities [5]. Therefore, it needs to be noted that a small proportion of MPA patients might present with PG patterns, especially nodules, which should be considered as systemic vasculitis manifestations clinically except for other possible causes.

It is noteworthy that airway lesions occurred among all groups. Airway-related abnormalities, including bronchiectasis, bronchial stenosis, tree-in-bud, and air trapping were all significantly common in the AI group. Moreover, air trapping and bronchial stenosis were both more frequent in the PG group than in the ILD group.

Of the 204 patients in the ILD group, UIP was the majority (71.6%), NSIP was noted in 39 (19.1%), OP was noted in 3 (1.5%), and unclassified IP was noted in 16 (7.8%).

### Predictors of mortality

For the whole cohort, infection was the most prevalent cause of death (39/66, 59.1%), active vasculitis only accounted for 19.7% (13/66) of the death. There were no statistical differences in the causes of death between the four groups (Additional file 1: Table S2).

**Fig. 3:**
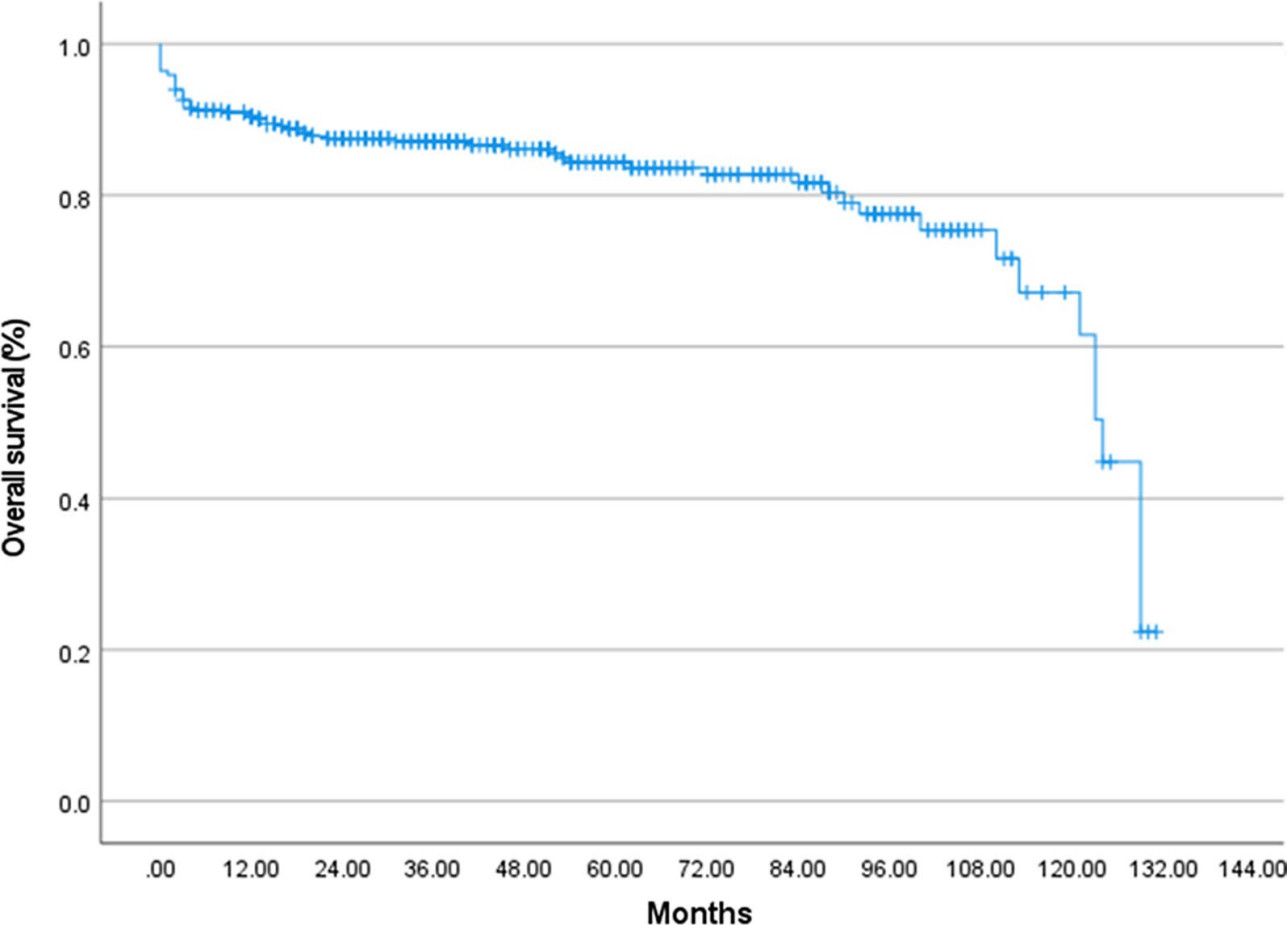
Kaplan–Meier survival curves for ANCA-associated vasculitis patients with pulmonary involvement

**Fig. 4:**
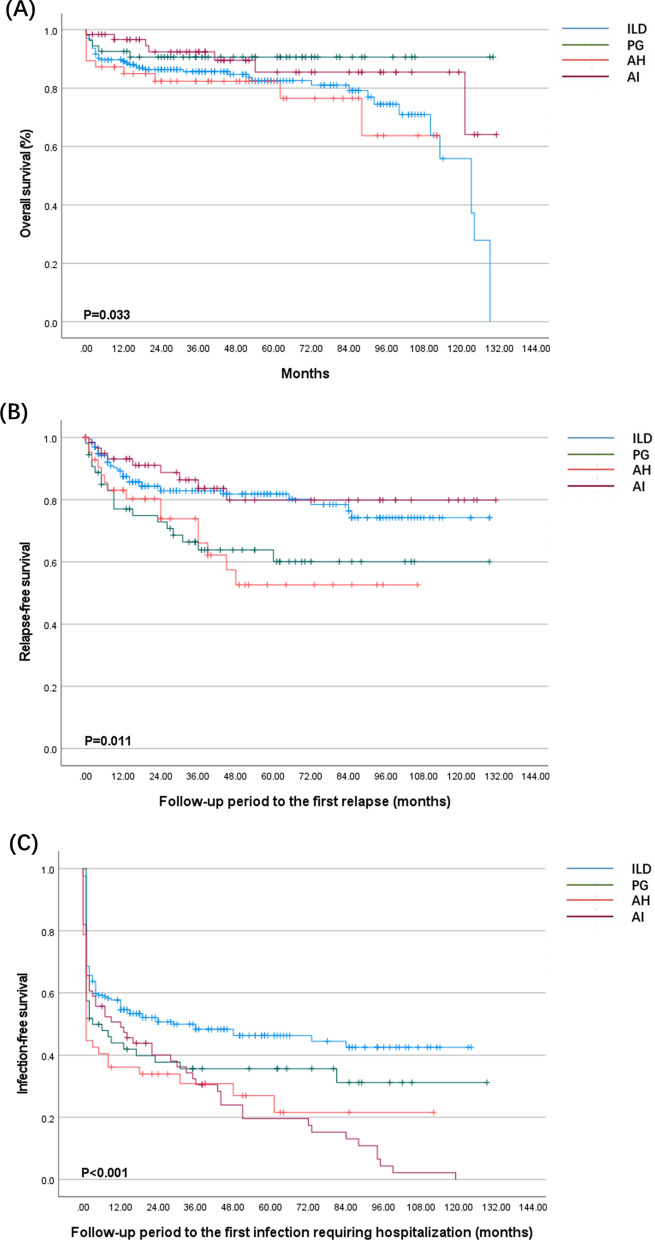
Kaplan–Meier analysis comparing ANCA-associated vasculitis patients with different patterns of pulmonary involvements. **a** overall survival; **b** relapse-free survival; **c** infection-free survival

During follow-up of a median duration of 42.0 months, 66 (18.0%) patients eventually died. The cumulative survival rate of overall patients at 1, 2, and 5 years was 90.4% (95% CI 88.9–91.9%), 87.5% (95% CI 85.7–89.3%) and 84.3% (95% CI 82.2–86.4%), respectively (Fig. 3). Survival was significantly different based on the radiologic features. One- and 5-year overall survival rates, respectively, were: 89.2% and 84.8% for ILD, 96.7% and 83.3% for AI, 85.1% and 83.0% for AH, 92.6% and 90.7% for PG (Fig. 4*p* = 0.033). Considering that there are fewer patients with OP patterns, we combined patients with OP and NSIP into a non-UIP subgroup to compare the prognosis between UIP and non-UIP groups. There was a significant difference in the overall survival between the UIP and non-UIP subgroup, patients with UIP patterns seemed to have a worse prognosis (Additional file 1: Fig. S1, *p* = 0.038).

In the multivariable Cox regression analysis, the independent predictors of all-cause mortality were UIP pattern, age ≥ 65 years at diagnosis, respiratory failure, infection requiring hospitalization, and AH (Table 2).

**Table 2:** Cox regression analysis of predictors for all-cause mortality in patients with ANCA-associated vasculitis with pulmonary involvement

	Univariate		Multivariate	
	Hazard ratio (CI 95%)	*p* value	Hazard ratio (CI 95%)	*p* value
*Radiological pattern*				
Interstitial lung disease	3.642 (1.318–10.066)	0.013		
UIP pattern	2.548 (1.515–4.284)	< 0.001	3.369 (1.922–5.906)	< 0.001
Pulmonary granuloma	0.484 (0.209–1.122)	0.091		
Alveolar hemorrhage	3.028 (1.757–5.218)	< 0.001	2.658 (1.491–4.738)	0.001
Airway involvement	1.806 (0.893 -3.650)	0.100		
Age ≥ 65 years at diagnosis	2.231 (1.237–4.024)	0.008	2.118 (1.151–3.896)	0.016
Sex (female)	1.156 (0.686–1.947)	0.587		
MPA	1.588 (0.752–3.354)	0.226		
GPA	0.619 (0.280–1.365)	0.234		
MPO-ANCA	2.349 (0.938–5.883)	0.068		
PR3-ANCA	0.756 (0.302–1.894)	0.551		
Cardiovascular comorbidities	1.240 (0.737–2.086)	0.418		
*Organ involvement at onset*				
Renal	1.733 (0.742–4.048)	0.204		
Ear, nose and throat	0.521 (0.285–0.954)	0.035		
Central nervous system	2.840 (1.217–6.629)	0.016		
Peripheral nervous system	1.213 (0.641–2.293)	0.553		
Cardiovascular	4.646 (1.131–19.085)	0.033		
Gastrointestinal	2.830 (1.024–7.823)	0.045		
Eye	0.596 (0.215–1.647)	0.318		
Respiratory failure	12.425 (7.036–21.944)	< 0.001	7.077 (3.731–13.423)	< 0.001
Initial serum creatine	1.001 (1.000–1.002)	0.082		
Renal insufficiency	1.518 (0.892–2.583)	0.123		
BVAS at onset	1.014 (0.989–1.041)	0.278		
*FFS (ref.* = *0)*				
FFS = 1	1.297 (0.758–2.219)	0.342		
FFS ≥ 2	1.112 (0.647–1.913)	0.700		
Infection requiring hospitalization	6.533 (2.977–14.337)	< 0.001	2.529 (1.042–6.137)	0.040
Relapse	1.010 (0.519–1.968)	0.976		
IS for induction therapy	0.617 (0.365–1.043)	0.071		
IS for maintenance therapy	1.560 (0.710–3.430)	0.269		

*ANCA* antineutrophil cytoplasm antibodies, *BVAS* Birmingham vasculitis activity scores, *FFS* five factors score, *GPA* granulomatosis with polyangiitis, *IS* immunosuppressant, *MPA* microscopic polyangiitis, *MPO* myeloperoxidase, *PR3* proteinase-3, *UIP* usual interstitial pneumonia

Given that the high proportion of ILD in our cohort, and the decrease of FVC and DLCO were reported as independent risk factors for mortality in connective tissue disease-related-ILD patients [24], we conducted survival analysis of the subgroup of cases with pulmonary functional results (Additional file 1: Table S3). The only factor significantly associated with shorter survival was DLCO decreased (HR 0.970, *p* = 0.019), and ROC curve analysis showed that the best cut-off value was 54.05% pred., with sensitivity and specificity of 66.7% and 63.4%, respectively (Additional file 1: Fig. S2).

### Predictors of relapse

During the follow-up, 111 (30.3%) patients experienced 186 relapses, all summarized in Table 3. The median duration from diagnosis to the first relapse was 12 [IQR 5–26] months. Pulmonary (59.7%) was the most prevalent organ involvement at the time of relapse, followed by the kidney (48.4%).

**Table 3:** Characteristics of relapses

	Alveolar hemorrhage (n = 47)	Interstitial lung disease (n = 204)	Pulmonary granuloma (n = 54)	Airway involvement (n = 61)
Relapse cases, no (%)	15/47 (31.9)	55/204 (27.0)	26/54 (48.1)^d^	15/61 (24.6)
Relapse events, no (%)	30	89	47	20
First relapsing time, median (IQR) (months)	13 (5–36)	12 (7–27)	9 (5–23)	9 (6–21)
*Organ involvement at relapse, no. (%)*				
Pulmonary	21/30 (70.0)	48/89(53.9)	35/47 (74.5)^d^	8/20 (40.0)
Renal	15/30 (50.0)	52/89 (58.4)^b^	12/47 (25.5)	11/20 (55.0)
General^a^	2/30 (6.7)	12/89 (13.5)	12/47 (25.5)	1/20 (5.0)
Ear, nose, and throat	2/30 (6.7)	2/89 (2.2)	4/47 (8.5)	6/20 (30.0)^e^
Central nervous system	0	2/89 (2.2)	9/47 (19.1)^b,c,d^	0
Peripheral nervous system	2/30 (6.7)	3/89 (3.4)	3/47 (6.4)	0
Gastrointestinal	1/30 (3.3)	1/89 (1.1)	3/47 (6.4)	3/20 (15.0)
Eye	2/30 (6.7)	5/89 (5.6)	3/47 (6.4)	1/20 (5.0)

*AH* Alveolar hemorrhage, *AI* Airway involvement, *ILD* interstitial lung disease, *PG* pulmonary granuloma

^a^General included fever or weight loss (≥ 2 kg)

*p* < 0.05: ^a^ILD versus PG, ^d^PG versus AH, ^e^PG versus AI, ^f^AH versus AI

The percentage of relapse cases was significantly higher in the PG group than in the AI group. Comparing organ involvements at relapse, pulmonary relapse was more common in the PG group than the AI group. Renal relapse was more common in the ILD group compared to the PG group. ENT relapse was more common in the AI group compared to the ILD group. CNS relapse was the most common in the PG group.

Relapse free–survival rates between the four groups were significantly different, patients with PG and AH were more likely to relapse (Fig. 4b *p* = 0.011). PR3-ANCA and CNS involvement were predictors of relapse in the multivariate Cox regression analysis (Additional file 1: Table S4). Moreover, we analyzed predictors of relapse in each group. Female, PR3-ANCA and CNS involvement were the predictors of relapse in the PG group. ENT involvement was the predictor of relapse in the AI group. Multivariate analysis did not yield a relevant clinical predictor of relapse in the ILD and AH groups (Additional file 1: Tables S5–S8).

Lastly, we investigated factors associated with pulmonary relapse, the leading organ involved at relapse. PG pattern and CNS involvement were risk factors of pulmonary relapse, but age ≥ 65 years was seen to be a protective factor (Additional file 1: Table S9).

### Predictors of infection

Since the infection was the leading cause of death in AAV patients with pulmonary involvement and also a significant factor independently associated with shorter survival, we further collected the infection-related data. There was a total of 347 infections in 237 patients during the follow-up. The median duration from diagnosis to the first infection was 3 [IQR 2–13] weeks. As shown in Table 4, pulmonary infection was the leading infection in each group. The genitourinary infection took second place.

**Table 4:** Details of infections during follow-up

	Alveolar hemorrhage (n = 47)	Interstitial lung disease (n = 204)	Pulmonary granuloma (n = 54)	Airway involvement (n = 61)
Infectious patients, no. (%)	34 (72.3)	105 (51.5)	43 (79.7)	55 (90.2)^a^
First in-patient infection time, median (IQR) (weeks)	2 (2–9)	3 (2–17)	3 (3–13)	3 (2–9)
Infectious episodes, no. (%)	53	147	60	87
*Infection pathogens, no. (%)*				
Bacteria	48/53 (90.6)	138/147 (93.8)	54/60 (90.0)	82/87 (94.3)
Fungus	20/53 (37.7)	38/147 (25.9)	15/60 (25.0)	20/87 (23.0)
*Pneumocystis jirovecii*	0	8/147 (5.4)	3/60 (5.0)	3/87 (3.4)
Aspergillus	3/53 (5.7)	6/147 (4.1)	6/60 (6.7)	3/87 (3.4)
Candida	12/53 (22.6)	24/147 (16.3)	8/60 (13.3)	16/87 (18.4)
Viral	6/53 (11.3)	17/147 (11.6)	6/60 (10.0)	4/87 (4.6)
CMV	2/53 (3.8)	6/147 (4.1)	6/60 (10.0)	1/87 (1.1)
EBV	1/53 (1.9)	4/147 (2.7)	4/60 (6.7)	0
Influenza virus	0	2/147 (1.4)	0	1/87 (1.1)
*Locations of infection, no. (%)*				
Pulmonary	50/53 (94.3)	135/147 (91.8)	57/60 (95.0)	85/87 (97.7)
Genitourinary	1/53 (1.9)	17/147 (11.7)	2/60 (3.3)	7/87 (8.0)
Gastrointestinal	4/53 (7.5)	5/147 (3.4)	2/60 (3.3)	2/87 (2.3)
Catheter-associated	1/53 (1.9)	4/147 (2.7)	1/60 (1.7)	2/87 (2.3)
Sepsis	1/53 (1.9)	8/147 (5.4)	2/60 (3.3)	0

*AH* Alveolar hemorrhage, *AI* Airway involvement, *CMV* cytomegalovirus, *EBV* Epstein-Barr virus, *ILD* interstitial lung disease, *PG* pulmonary granuloma

*p* < 0.05: ^a^ILD vs. AI

Regarding the pathogens of infection, the bacterium was the most common pathogen (92.8%), followed by fungus and virus (26.8%, and 9.2%, respectively). Candida was the most frequent fungal infection (17.3%), followed by Aspergillus (5.2%). *Pneumocystis jirovecii* was found in 14 cases (4.0%), 8 from the ILD group and 3 from PG and AI group respectively, leading to 9 deaths in total (6 in the ILD group, 1 in the PG group, and 2 in the AI group). Cytomegalovirus (4.3%) ranked first among viral pathogens, followed by Epstein–Barr virus and influenza virus (2.6%, and 0.9%, respectively).

The percentage of infectious patients in the AI group was higher than that in the ILD group. However, there were no significant differences regarding the infection pathogens and locations between the four groups.

The infection-free survival curve was especially steep within the first 12 months, especially the first 3 months. Three- and twelve-month infection-free survival was 55.7% and 48.4%. There was a significant difference in the infection-free survival between the four groups (Fig. 4c *p* < 0.001). Multiple Cox regression analysis showed that the following factors were independent predictors of secondary infections: respiratory failure, AI, AH, and glucocorticoids pulse for induction therapy (Additional file 1: Table S10). Immunosuppressors for both induction and maintenance therapy did not significantly associate with infection.

## Discussion

To the best of our knowledge, this is currently the largest Chinese cohort focused on AAV-related pulmonary involvement with a long follow-up period and is therefore representative. We only enrolled the AAV patients with pulmonary involvement in this cohort to comprehensively clarify the clinical characteristics of these patients and if different radiological subgroups had differences in clinical features, survival, the risk of both relapse and secondary infection.

Firstly, our cases are older compared to the previous European studies and roughly the same age as other Asian cohorts, but these studies only focused on AAV patients with renal involvement [25–27]. Compared to the studies focusing on AAV-related lung involvement, similar characteristics are also observed, probably due to a higher proportion of ILD, especially UIP pattern, in Asian AAV patients [4–6]. Secondly, the ILD group had a lower BVAS, which was in contrast to a previous Japanese study [4]. This indicated the vasculitis activities in AAV-related ILD patients are relatively low at diagnosis, careful dosing of immunosuppressor is called for induction treatment by weighing the infectious side effect. Finally, this is the first study to document the bronchoalveolar lavage and pulmonary function results of patients with AAV-related pulmonary involvement in detail, confirming that these results differ based on the radiological images and that DLCO might partially predict patient outcomes. Given that patients with PG are relatively rare and partially similar to the radiological pattern of lung cancer [28, 29], the clinical utility of transbronchial lung biopsy at the time of AAV diagnosis should be considered. In summary, we observe that patients with different types of lung involvement have unique clinical characteristics.

As for radiological features, our study highlights the importance of AI in AAV patients, and further determines the global pattern of abnormalities in ILD patients. In our cohort, 16.7% of patients (AI group) presented with lower respiratory tracts involvement alone, which is second only to the ILD. However, a study by Yamagata et al. showed that airway lesions were as common as ILD, accounting for 66% [3]. This discrepancy may be explained by the fact that our study excluded ILD-related traction bronchiectasis as well as airway lesions due to other comorbidities. A few studies have focused on the association between AI and AAV, suggesting that AI is comorbidity rather than one of the pulmonary manifestations of AAV [30, 31]. Conversely, some patients with AI substantially improved after the initial immunosuppressive treatment in our study, therefore we tend to consider AI as a part of systemic manifestation. Specifically, AAV-related AI is more frequently characterized by small airway disease. Pulmonary functional results further confirm these radiologic findings, since FEF_25–75_ and FEF_50_, widely regarded as markers of small airways obstruction, are significantly decreased in the AI and PG groups. Recent data suggest that the airway could be a site of initiation of immune response in AAV, which might correlate with neutrophil activation [31, 32]. Correspondingly, our study reports that neutrophils count is relatively higher in bronchoalveolar lavage fluids of patients from the AI group.

Another innovation point lies in the careful classification of all ILD patients. Our data show that UIP account for 39.9% of the total patients with AAV-related lung involvement. As for the ILD subgroup, UIP accounts for 71.6%, which is significantly higher than the previous studies (38–57%) [6, 8, 33]. NSIP is found in only 19.1% of ILD patients, which is much lower than a European study [7]. Meanwhile, 3 cases presented as OP, 2 of them with MPO-ANCA positive and 1 with PR3-ANCA positive, which is a rare and unique imaging manifestation of AAV, with only a few previous cases reported [34–36]. Hence, our study suggests that the imaging findings of AAV can be diverse and OP can be a rare manifestation of AAV-related ILD.

Although pulmonary involvement is known as a predictor of poor prognosis of AAV, the patients in our pulmonary-involvement cohort seemed to have a better prognosis than previous reports. The median duration of follow-up of our study was 42.0 months and 66 (18.0%) deaths were recorded. The 1-year and 5-year survival rates are 90.4% and 84.3% respectively. The previous study mostly by nephrologists and rheumatologists reported that the cumulative survival was 77.4–88% and 61.9–78% at 1 and 5 years [2, 9, 25–27]. As same as Flossmann’s report [26], infection and active vasculitis were the main cause of the 1-year death in our cohort. Furthermore, we also found the independent predictors of all-cause mortality during long-term follow-up in AAV patients with pulmonary involvement were UIP pattern, age ≥ 65 years at diagnosis, respiratory failure, infection needing hospitalization and AH, but not including initial renal function, higher BVAS, lower hemoglobin, and higher white cell count in other publications [9, 25–27, 30]. This may be partly explained by the specificity of our cohort. Firstly, the patients without lung involvement were excluded, which might remove some patients with other important organ injuries and high BVAS, such as those with more severe renal failure. Secondly, we enrolled the patients in the last decade with more standardized treatment and biological agents which gave them more opportunities.

Survival is significantly different based on the radiologic features. ILD, especially UIP pattern, and AH groups tend to have a poor prognosis. AH is associated with early prognosis whereas ILD is related to long-term outcome, which is in line with a previous Japanese study [4]. Our cohort is a representative Chinese cohort with updated treatment and longer follow-up duration than that in the previous study. In addition to the ILD patients who died from respiratory failure, some also died from cardiovascular events. A recent meta-analysis reported that excess mortality due to cardiovascular events ranges from about two to fourfold in AAV and is higher in the elderly [37]. Patients with AI have a better early prognosis but a decreasing trend in the long-term outcome, probably due to an increased risk of infection during follow-up. Therefore, carefully identifying the baseline chest imaging characteristics of AAV patients can predict their prognosis.

Relapse is another major concern. Pulmonary involvement is regarded as a risk factor of relapse [1]. However, no study pays attention to the specific relapse events among these patients. Beyond that, the value of measuring ANCA to predict a relapse is doubtable. Kemna et al. reported that an ANCA rise was related to a relapse only in patients with renal involvement [38]. Our study reveals that AAV patients with lung involvement have a significantly higher risk of relapse than previously reported in patients with renal involvement [39]. Comparing subgroups, relapse is more frequent in the PG and AH groups and less common in the ILD and AI groups. Regarding the relapse organs, patients in the PG and AH groups are more likely to have a pulmonary relapse, whereas patients in the ILD and AI groups are more prone to renal relapse. We further identify that PR3-ANCA positive and CNS involvement are risk factors of relapse in AAV-related lung involvement patients. Moreover, predictors of relapse are distinct in each imaging subgroup. Of note, PG pattern is an independent risk factor for pulmonary relapse. This is the first study to elaborate on the relapse of AAV-related lung involvement patients and to demonstrate that there is a correlation between relapse and imaging patterns.

Infection was the leading cause of death in AAV patients, and underlying pulmonary involvement was regarded as an independent predictor of secondary infection [25, 40].In the present study, more than half of the patients died from infection. Three- and twelve-month infection-free survival is 55.7% and 48.4%, which is significantly higher than a previous Chinese study focusing on AAV patients with renal involvement [25]. Regarding the infectious location, pulmonary accounts for more than 90% of the infection. Fungal and viral infections are not uncommon due to active immunosuppressive therapy. Multivariate analysis reveals that AH and AI are both independently related to a higher risk of infection. While AI patients in this study are less prone to relapse. Therefore, immunosuppressive therapy strategies must keep into account not only AAV activity but patients’ radiological characteristics and their risk of infection as well.

In addition, the first infection time was significantly earlier in our study compared to others [2, 41], which focused on patients with AAV. Whereas, our study is a large-scale cohort of AAV patients with pulmonary involvement. On the one hand, this confirmed that pulmonary involvement is a contributing factor to the high risk of infection in patients with AAV. On the other hand, we firstly reported that the first infection time was significantly earlier among AAV patients with pulmonary involvement, with a median interval of only 3 weeks from diagnosis to the first infection. Some studies have focused on the close relationship between infection and AAV, suggesting that long-term chronic infection might be one of the mechanisms of ANCA production and AAV pathogenesis [42, 43]. Moreover, a recent study reported that AAV patients have IFN (type I interferon) system dysfunction, thus leading to patients’ long-term susceptibility to infection [44]. Therefore, the preventive method should be employed among these AAV patients with pulmonary involvement, such as administration of trimethoprim-sulfamethoxazole for *Pneumocystis jirovecii* prophylaxis.

There were some limitations in our study. On the one hand, ours is a retrospective study from a single center and therefore may be affected by referral bias and missing data, including histopathological data, which are the diagnostic gold standards of different patterns of pulmonary lesions. Therefore, we call for the increasing clinical utility of transbronchial lung biopsy among these subjects. On the other hand, it was not possible to determine whether preexisting AI was associated with AAV, we thus excluded these patients. Nevertheless, a recent study reported that the prognosis of patients with bronchiectasis prior to AAV diagnosis is poorer, which needs further investigation [32].

## Conclusion

This study demonstrates that AI is also one of the common radiological manifestations of AAV, and AAV-related pulmonary involvement with diverse radiological features have different clinical characteristics and outcome. Specifically, the ILD group tends to have a poor long-term prognosis, the PG group is prone to relapse, and the AI group is apt to infection. The AH group has a high risk of both early infection and relapse, thus a poor short-term prognosis. Therefore, the intensity of immunosuppressive therapy must be carefully valued by considering the baseline CT findings among AAV patients with pulmonary involvement.

## Supplementary Information

**Additional file 1**. Supplementary information for the manuscript.

## Data Availability

The datasets used and/or analyzed during the current study are available from the corresponding author on reasonable request.

## Abbreviations

AAV: Antineutrophil cytoplasm antibodies-associated vasculitis
AI: Airway involvement
ANCA: Antineutrophil cytoplasm antibodies
BVAS: Birmingham vasculitis activity scores
CNS: Central nervous system
CT: Computed tomography
DLCO: Diffusing capacity for carbon monoxide
EGPA: Eosinophilic granulomatosis with polyangiitis
ENT: Ear-nose-throat
FEF_25–75_: Forced expiratory flow at 25–75% of FVC
FEF_50_: Forced expiratory flow at 50% of FVC
FEF_25_: Forced expiratory flow at 50% of FVC
FEV1: Forced expiratory volume
FFS: Five factors score
FVC: Forced vital capacity
GPA: Granulomatosis with polyangiitis
MPA: Microscopic polyangiitis; no: number
no: Number
MPO: Myeloperoxidase
NSIP: Non-specific interstitial pneumonia
OP: Organizing pneumonia
PR3: Proteinase-3
TLC: Total lung capacity
Pred.: Predicted
UIP: Usual interstitial pneumonia
