# Supplementary material for: Pulmonary involvement of ANCA-associated vasculitis in adult Chinese patients
Source: BMC Pulm Med. 2022 Jan 12;22:35. doi: 10.1186/s12890-022-01829-y (PMC8756656; doi:10.1186/s12890-022-01829-y)
Supplement: Supplementary file 1 — Additional file 1. Supplementary information for the manuscript. [file 12890_2022_1829_MOESM1_ESM.docx]

Additional file 1: Table S1. Prevalence of individual components of pulmonary abnormalities on chest CT.

| **Abnormality, no. (%)** | **Alveolar hemorrhage (n=47)** | **Interstitial lung disease (n=204)** | **Pulmonary granuloma (n=54)** | **Airway involvement**  **(n=61)** |
| --- | --- | --- | --- | --- |
| **Interstitial lung disease** | | | | |
| Ground glass opacity | 29(61.7) | 110(53.9) | 26(48.1) | 30(49.2) |
| Honeycombing | 3(6.4) | 139(68.1) ^a,b,c^ | 1(1.9) | 3(4.9) |
| Reticulation | 5(10.6) | 36(17.6) ^a^ | 1(1.9) | 5(8.2) |
| ILD classification | | | | |
| UIP | - | 146(71.6) | - | - |
| NSIP | - | 39(19.1) | - | - |
| COP | - | 3(1.5) | - | - |
| Unclassified IP | - | 16(7.8) | - | - |
| **Pulmonary granuloma** | | | | |
| Nodules | 0 | 2(1.0) | 25(46.3) ^a,d,e^ | 1(1.6) |
| Masses | 2(4.3) | 3(1.5) | 27(50.0) ^a,d,e^ | 2(3.3) |
| Cavities | 1(2.3) | 1(0.5) | 18(33.3) ^a,d,e^ | 2(3.3) |
| **Airway involvement** | | | | |
| Bronchiectasis ^1^ | 8(17.0) | 20(9.8) | 11(20.4) | 29(47.5) ^c,e,f^ |
| Bronchial stenosis | 6(12.8) | 12(5.9) ^a^ | 13(23.7) | 21(34.4) ^c^ |
| Tree-in-bud | 5(10.6) | 8(3.9) | 7(13.0) | 24(39.3) ^c,e,f^ |
| Air trapping | 8(17.0) | 9(4.4) ^a,b^ | 9(16.7) | 35(57.4) ^c,e,f^ |
| **Other** | | | | |
| Emphysema | 4(8.5) | 11(5.4) | 0 | 0 |
| Pleural thickening | 1(2.3) | 3(1.5) | 1(1.9) | 1(1.6) |

AH: alveolar hemorrhage; AI: airway involvement; COP: cryptogenic organizing pneumonia; CT: computed tomography; ILD: interstitial lung disease; NSIP: non-specific interstitial pneumonia; PG: pulmonary granuloma; UIP: usual interstitial pneumonia.

^1^ Bronchiectasis excluded the ILD-related traction bronchiectasis.

p< 0.05: ^a^ ILD vs. PG, ^b^ ILD vs. AH, ^c^ ILD vs. AI, ^d^ PG vs. AH, ^e^ PG vs. AI, ^f^ AH vs. AI

Additional file 1: Table S2. Outcome of patients with different radiological patterns.

|  | **Alveolar hemorrhage (n=47)** | **Interstitial lung disease (n=204)** | **Pulmonary granuloma (n=54)** | **Airway involvement**  **(n=61)** |
| --- | --- | --- | --- | --- |
| Respiratory failure, no. (%) | 16(34.0) | 63(30.9) | 8(14.8) | 12(19.7) |
| Intensive Care Unit, no. (%) | 16(34.0) | 35(17.2) | 2(3.7) ^d^ | 9(14.8) |
| Death, no. (%) | 10(21.3) | 44(21.6) | 5(9.3) | 7(11.5) |
| Infection | 6(12.8) | 24(11.8) | 3(5.6) | 6(9.8) |
| Active AAV | 4(8.5) | 6(2.9) | 2(3.7) | 1(1.6) |
| Cardiovascular events | 0 | 2(4.3) | 0 | 0 |
| Unknown | 0 | 12(5.8) | 0 | 0 |

p< 0.05: ^d^ PG vs. AH

Additional file 1: Figure S1: Overall survival of patients with AAV-UIP and non-UIP patterns (p=0.038). AAV: ANCA-associated vasculitis; UIP: usual interstitial pneumonia.


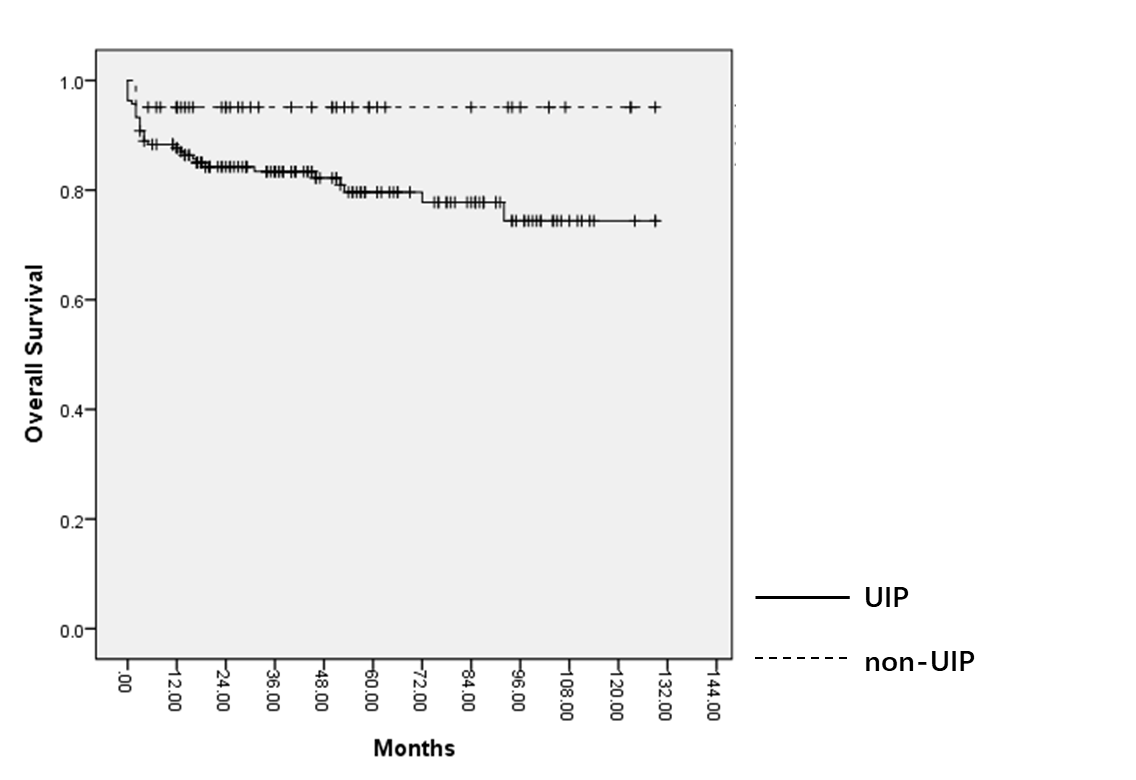


Additional file 1: Table S3: Univariate analysis of pulmonary functional results on patient’s survival (n=106).

|  | **Univariate** | |
| --- | --- | --- |
| Pulmonary function index | Hazard ratio (CI 95%) | p value |
| FEV1/FVC | 0.976(0.948-1.005) | 0.111 |
| FEV1 % pred. | 0.985(0.967-1.004) | 0.122 |
| FVC% pred. | 0.989(0.958-1.021) | 0.481 |
| TLC% pred. | 0.987(0.964-1.010) | 0.268 |
| FEF 25-75% pred. | 0.939(0.869-1.014) | 0.108 |
| FEF50% pred. | 1.028(0.975-1.084) | 0.306 |
| FEF25% pred. | 1.018(0.994-1.042) | 0.140 |
| DLCO % pred. | 0.970(0.945-0.995) | 0.019 |

AAV: ANCA-associated vasculitis; DLCO: diffusing capacity for carbon monoxide; FEF _25-75_=forced expiratory flow at 25–75% of FVC; FEF _50_=forced expiratory flow at 50% of FVC; FEF _25_=forced expiratory flow at 50% of FVC; FEV1: forced expiratory volume; FVC: forced vital capacity; TLC: total lung capacity; % pred: % predicted.

Additional file 1: Figure S2: Receiver-operating characteristic (ROC) curves for the diffusing capacity for carbon monoxide (DLCO) predicted mortality.


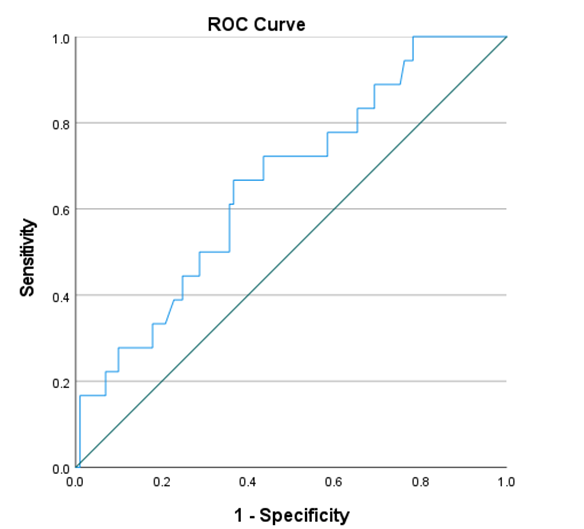


The area under the curve was 0.660 (95% CI 0.530 to 0.791, p=0.031) and the best cut-off value was 54.05%, with sensitivity and specificity of 66.7% and 63.4%, respectively.

Additional file 1: Table S4. Cox regression analysis of predictors for relapse.

|  | **Univariate** | | **Multivariate** | |
| --- | --- | --- | --- | --- |
|  | Hazard ratio (CI 95%) | p value | Hazard ratio (CI 95%) | p value |
| Age≥65 years | 1.103(0.701-1.737) | 0.671 |  |  |
| Sex (female) | 0.765(0.525-1.116) | 0.165 |  |  |
| MPO | 0.610(0.379-0.939) | 0.025 |  |  |
| PR3 | 2.322(1.439-3.746) | 0.001 | 2.387(1.477-3.857) | <0.001 |
| BVAS | 1.003(0.983-1.023) | 0.776 |  |  |
| Interstitial lung disease | 0.666(0.424-1.046) | 0.078 |  |  |
| Pulmonary granuloma | 1.766(1.041-2.994) | 0.035 |  |  |
| Airway involvement | 0.597(0.298-1.196) | 0.146 |  |  |
| Alveolar hemorrhage | 1.895(1.078-3.330) | 0.026 |  |  |
| **Organ involvement at onset** | | | | |
| Renal | 1.102(0.667-1.821) | 0.704 |  |  |
| Ear, nose and throat | 1.228(0.839-1.797) | 0.291 |  |  |
| Central nervous system | 2.543(1.230-5.256) | 0.012 | 2.700 (1.304-5.590) | 0.007 |
| Peripheral nervous system | 0.821(0.494-1.365) | 0.447 |  |  |
| Eye | 1.109(0.620-1.984) | 0.728 |  |  |
| GCs pulse for induction therapy | 1.269(0.867-1.857) | 0.221 |  |  |
| PEX for induction therapy | 0.840(0.524-1.345) | 0.468 |  |  |
| IS for induction therapy | 1.370(0.665-2.822) | 0.394 |  |  |
| IS for maintenance therapy | 2.852(0.901-9.021) | 0.075 |  |  |

BVAS: Birmingham Vasculitis Activity Scores; GCs: glucocorticoids; IS: immunosuppressant; MPA: microscopic polyangiitis; MPO: myeloperoxidase; PEX: plasma exchange; PR3: proteinase-3; UIP: usual interstitial pneumonia.

Additional file 1: Table S5. Cox regression analysis of predictors for relapse in AAV-ILD patients.

|  | **Univariate** | | **Multivariate** | |
| --- | --- | --- | --- | --- |
|  | Hazard ratio (CI 95%) | p value | Hazard ratio (CI 95%) | p value |
| Age≥65 years | 0.955(0.958-1.013) | 0.729 |  |  |
| Sex (female) | 0.823(0.329 -1.729) | 0.607 |  |  |
| MPO | 0.318(0.096 -1.050) | 0.060 |  |  |
| PR3 | 1.463 (0.484-4.419) | 0.500 |  |  |
| BVAS | 1.029 (0.915-1.157) | 0.639 |  |  |
| UIP pattern | 1.091 (0.471 -2.526) | 0.839 |  |  |
| **Organ involvement at onset** | | | | |
| Renal | 0.528(0.092 -3.025) | 0.437 |  |  |
| Ear, nose and throat | 0.787(0.270-2.292) | 0.662 |  |  |
| Central nervous system | 2.490(0.508-12.213) | 0.261 |  |  |
| Peripheral nervous system | 0.306(0.067-1.407) | 0.128 |  |  |
| Eye | 0.974 (0.232 -4.080) | 0.971 |  |  |
| GCs pulse for induction therapy | 1.327(0.614-2.869) | 0.472 |  |  |
| PEX for induction therapy | 1.694 (0.702-4.087) | 0.241 |  |  |
| IS for induction therapy | 1.252(0.542-2.895) | 0.599 |  |  |
| IS for maintenance therapy | 2.043(0.639-6.530) | 0.228 |  |  |

AAV: ANCA-associated vasculitis; BVAS: Birmingham Vasculitis Activity Scores; GCs: glucocorticoids; ILD: interstitial lung disease; IS: immunosuppressant; MPA: microscopic polyangiitis; MPO: myeloperoxidase; PEX: plasma exchange; PR3: proteinase-3; UIP: usual interstitial pneumonia.

Additional file 1: Table S6. Cox Regression Analysis of Predictors for Relapse in AAV-PG patients.

|  | **Univariate** | | **Multivariate** | |
| --- | --- | --- | --- | --- |
|  | Hazard ratio (CI 95%) | p value | Hazard ratio (CI 95%) | p value |
| Age≥65 years | 0.712 (0.325-1.561) | 0.396 |  |  |
| Sex (female) | 2.524(1.172-5.434) | 0.018 | 2.593(1.153-5.833) | 0.021 |
| MPO | 0.441(0.205-0.948) | 0.036 |  |  |
| PR3 | 2.797(1.340-5.837) | 0.006 | 2.416 (1.130-5.167) | 0.023 |
| BVAS | 1.011(0.975-1.047) | 0.560 |  |  |
| **Organ involvement at onset** | | | | |
| Renal | 0.926 (0.375-2.284) | 0.867 |  |  |
| Ear, nose and throat | 1.797(0.798-4.048) | 0.157 |  |  |
| Central nervous system | 5.577(1.904-16.335) | 0.002 | 7.965 (2.562-24.758) | <0.001 |
| Peripheral nervous system | 1.029(0.356-2.969) | 0.958 |  |  |
| Eye | 1.034(0.441-2.421) | 0.939 |  |  |
| GCs pulse for induction therapy | 1.059(0.513-2.186) | 0.877 |  |  |
| PEX for induction therapy | 0.874(0.354-2.154) | 0.769 |  |  |
| IS for induction therapy | 1.935(0.459-8.157) | 0.368 |  |  |

AAV: ANCA-associated vasculitis; BVAS: Birmingham Vasculitis Activity Scores; GCs: glucocorticoids; IS: immunosuppressant; MPA: microscopic polyangiitis; MPO: myeloperoxidase; PEX: plasma exchange; PG: pulmonary granuloma; PR3: proteinase-3.

Additional file 1: Table S7. Cox Regression Analysis of Predictors for Relapse in AAV-AI patients.

|  | **Univariate** | | **Multivariate** | |
| --- | --- | --- | --- | --- |
|  | Hazard ratio (CI 95%) | p value | Hazard ratio (CI 95%) | p value |
| Age≥65 years | 0.966(0.918-1.016) | 0.177 |  |  |
| Sex (female) | 0.304 (0.076-1.219) | 0.093 |  |  |
| MPO | 0.168 (0.033-0.865) | 0.033 |  |  |
| PR3 | 5.394(1.332-21.839) | 0.018 |  |  |
| BVAS | 1.010(0.954-1.070) | 0.720 |  |  |
| **Organ involvement at onset** | | | | |
| Renal | 0.498(0.102-2.433) | 0.389 |  |  |
| Ear, nose and throat | 10.493(1.312-83.929) | 0.027 | 9.276(1.083-79.436) | 0.042 |
| Central nervous system | 7.460(0.829-67.152) | 0.073 |  |  |
| Peripheral nervous system | 0.326(0.041-2.614) | 0.291 |  |  |
| Eye | 1.188(0.148-9.505) | 0.871 |  |  |
| GCs pulse for induction therapy | 2.704(0.561-13.039) | 0.215 |  |  |
| PEX for induction therapy | 0.796(0.165-3.844) | 0.777 |  |  |
| IS for induction therapy | 23.100(0.001-650482.724) | 0.548 |  |  |

AAV: ANCA-associated vasculitis; AI：airway involvement; BVAS: Birmingham Vasculitis Activity Scores; GCs: glucocorticoids; IS: immunosuppressant; MPA: microscopic polyangiitis; MPO: myeloperoxidase; PEX: plasma exchange; PG: pulmonary granuloma; PR3: proteinase-3.

Additional file 1: Table S8. Cox Regression Analysis of Predictors for Relapse in AAV-AH patients.

|  | **Univariate** | | **Multivariate** | |
| --- | --- | --- | --- | --- |
|  | Hazard ratio (CI 95%) | p value | Hazard ratio (CI 95%) | p value |
| Age≥65 years | 0.979(0.943-1.016) | 0.265 |  |  |
| Sex (female) | 2.565(0.722-9.105) | 0.145 |  |  |
| MPO | 1.631(0.367-7.249) | 0.520 |  |  |
| PR3 | 0.539(0.122-2.391) | 0.416 |  |  |
| BVAS | 1.003(0.955-1.053) | 0.915 |  |  |
| **Organ involvement at onset** | | | | |
| Renal | 21.055(0.000-254652317.2) | 0.714 |  |  |
| Ear, nose and throat | 0.995(0.339-2.920) | 0.993 |  |  |
| Central nervous system | 1.265(0.166-9.657) | 0.821 |  |  |
| Peripheral nervous system | 0.042(0.000-78.448) | 0.409 |  |  |
| Eye | 1.706(0.479-6.079) | 0.410 |  |  |
| GCs pulse for induction therapy | 1.728(0.487-6.131) | 0.398 |  |  |
| PEX for induction therapy | 0.662(0.225-1.949) | 0.454 |  |  |
| IS for induction therapy | 24.201(0.016-37575.092) | 0.395 |  |  |

AAV: ANCA-associated vasculitis; AH: alveolar hemorrhage; BVAS: Birmingham Vasculitis Activity Scores; GCs: glucocorticoids; IS: immunosuppressant; MPA: microscopic polyangiitis; MPO: myeloperoxidase; PEX: plasma exchange; PG: pulmonary granuloma; PR3: proteinase-3.

Additional file 1: Table S9. Cox regression analysis of predictors for pulmonary relapse.

|  | **Univariate** | | **Multivariate** | |
| --- | --- | --- | --- | --- |
|  | Hazard ratio (CI 95%) | p value | Hazard ratio (CI 95%) | p value |
| Age≥65 years | 0.578(0.396-0.845) | 0.005 | 0.642(0.436-0.944) | 0.024 |
| Sex (female) | 1.375(0.822-2.298) | 0.225 |  |  |
| MPO | 0.610(0.397-0.939) | 0.025 |  |  |
| PR3 | 2.322(1.439-3.746) | 0.001 |  |  |
| BVAS | 1.023(0.997-1.048) | 0.079 |  |  |
| Interstitial lung disease | 1.463(0.878-2.438) | 0.144 |  |  |
| Pulmonary granuloma | 2.071(1.381-3.107) | <0.001 | 1.910 (1.264-2.866) | 0.002 |
| Airway involvement | 1.568(0.626-3.930) | 0.337 |  |  |
| Alveolar hemorrhage | 1.189(0.578-2.449) | 0.638 |  |  |
| **Organ involvement at onset** | | | | |
| Renal | 0.637(0.349-1.162) | 0.142 |  |  |
| Ear, nose and throat | 1.451(0.873-2.411) | 0.151 |  |  |
| Central nervous system | 2.543(1.230-5.256) | 0.012 | 2.514 (1.215-5.200) | 0.013 |
| Peripheral nervous system | 0.501(0.215-1.164) | 0.108 |  |  |
| Eye | 1.246(0.591-2.623) | 0.563 |  |  |
| GCs pulse for induction therapy | 1.589(0.957-2.636) | 0.073 |  |  |
| PEX for induction therapy | 0.883(0.485-1.609) | 0.685 |  |  |
| IS for induction therapy | 0.955(0.411-2.221) | 0.915 |  |  |
| IS for maintenance therapy | 2.159(0.527-8.843) | 0.285 |  |  |

BVAS: Birmingham Vasculitis Activity Scores; GCs: glucocorticoids; IS: immunosuppressant; MPA: microscopic polyangiitis; MPO: myeloperoxidase; PEX: plasma exchange; PR3: proteinase-3; UIP: usual interstitial pneumonia.

Additional file 1: Table S10. Cox regression analysis of predictors for secondary infection.

|  | **Univariate** | | **Multivariate** | |
| --- | --- | --- | --- | --- |
|  | Hazard ratio (CI 95%) | p value | Hazard ratio (CI 95%) | p value |
| Age≥65 years | 1.091(0.829-1.435) | 0.534 |  |  |
| Sex (female) | 1.042(0.796-1.364) | 0.766 |  |  |
| MPO | 0.965(0.680-1.371) | 0.844 |  |  |
| PR3 | 1.245(0.836-1.854) | 0.280 |  |  |
| BVAS | 1.016(1.003-1.030) | 0.017 |  |  |
| Interstitial lung disease | 1.031(0.725-1.464) | 0.867 |  |  |
| UIP pattern | 1.171(0.679-2.017) | 0.570 |  |  |
| Pulmonary granuloma | 1.175(0.816-1.692) | 0.386 |  |  |
| Airway involvement | 1.165(1.216-2.241) | 0.001 | 1.943(1.402-2.694) | <0.001 |
| Alveolar hemorrhage | 1.684(1.165-2.435) | 0.006 | 1.852(1.257-2.728) | 0.002 |
| Respiratory failure | 2.978(2.323-3.972) | <0.001 | 2.875(2.150-3.846) | <0.001 |
| GCs pulse for induction therapy | 1.831(1.254-2.673) | 0.002 | 1.588(1.083-2.326) | 0.018 |
| PEX for induction therapy | 1.641(1.089-2.472) | 0.018 |  |  |
| IS for induction therapy | 0.862(0.568-1.309) | 0.486 |  |  |
| IS for maintenance therapy | 1.062(0.628-1.796) | 0.822 |  |  |

BVAS: Birmingham Vasculitis Activity Scores; GCs: glucocorticoids; IS: immunosuppressant; MPA: microscopic polyangiitis; MPO: myeloperoxidase; PEX: plasma exchange; PR3: proteinase-3; UIP: usual interstitial pneumonia.
